# Over-expression of CCK1 Receptor Reverse Morphine Dependence

**DOI:** 10.1007/s10989-018-9696-7

**Published:** 2018-04-02

**Authors:** Lijing Hao, Di Wen, Hongyan Gou, Feng Yu, Bin Cong, Chunling Ma

**Affiliations:** 1 Department of Forensic Medicine, Hebei Key Laboratory of Forensic Medicine, Collaborative Innovation Center of Forensic Medical Molecular Identification, Hebei Medical University, Shijiazhuang, 050017 Hebei Province People’s Republic of China; 2 Department of Anesthesiology, The Third Hospital of Hebei Medical University, 139 Ziqiang Road, Shijiazhuang, 050051 People’s Republic of China; 3 CUHK Shenzhen Research Institute, 2 Yuexing Road, Nanshan District, Shenzhen, 518057 People’s Republic of China

**Keywords:** Morphine, µ-Opioid receptor, CCK receptor, cAMP, CREB, ERK

## Abstract

Studies demonstrated that cholecystokinin (CCK) system involved in morphine dependence and withdrawal. Our previous study showed that endogenous CCK system were up-regulated after chronic morphine exposure. Additionally, CCK1 receptor significantly blocked the inhibitory effect of exogenous CCK-8 on morphine dependence, but CCK2 receptor appears to be necessary for low concentrations of endogenous CCK to potentiate morphine dependence. Therefore, CCK1R and CCK2R function differently in chronic morphine dependence, but the mechanism is still unclear. In this study, HEK-293 cells co-transfected with µ-opioid receptors (HEK293-hMOR) and CCK1R or CCK2R were established. Cells were treated with 10 µM morphine for 6, 12, 16, 24 h and 100 µM naloxone precipitation for 15 min. cAMP overshoot was appeared at 12 h and was increased time dependently after morphine exposure in HEK293-hMOR cells. The cAMP overshoot did not appear in CCK1R-overexpressing HEK293-hMOR cells, while still appeared in CCK2R-overexpressing HEK293-hMOR cells. Over-expression of CCK1R reversed CREB and ERK1/2 activation in HEK293-hMOR cells exposed to morphine. Our study identifies over-expression of CCK1R significantly blocked morphine dependence, which was related with phosphorylation of CREB, and ERK1/2 signaling activation. While over-expression of CCK2R promoted morphine dependence, which was related with phosphorylation of CREB but not ERK1/2 signaling activation.

## Introduction

Opioids, such as morphine, are well known for their analgesic effects and addictive properties. Chronic use of opioids results in the development of dependence, leading to a withdrawal syndrome upon abrupt discontinuation (Williams et al. 2013), which greatly limits their clinical use. While cholecystokinin peptides (CCK) have been shown involved in many opioid-mediated effects. Morphine treatment enhances CCK overflow (de Araujo Lucas et al. 1998; Zhou et al. 1992) whereas CCK-8 and its analogues attenuate the analgesic effects of morphine(Faris 1985; Faris et al. 1983; Han et al. 1986). The administration of CCK receptor antagonists can prevent or reverse tolerance to systemic exogenous opioids or electroacupuncture-induced analgesia, and suppress morphine withdrawal syndrome (Felicio et al. 2001; Han et al. 1985, 1986; Lu et al. 2001; Mitchell et al. 2006; Valverde and Roques 1998; Wen et al. 2012a; Xiong and Yu 2006). CCK receptors are classified into two subtypes, CCK1R and CCK2R, based on their pharmacological properties and distribution of specific ligand binding sites. Previous studies have used different receptor antagonists to study the role of the two receptor subtypes and revealed they have different roles in the central nervous system. Lu et al. concluded that CCK1R present in discrete regions of the brain has a low affinity for central CCK and is not involved in the development of morphine tolerance and dependence (Lu et al. 2001). However, we previously found that chronic pretreatment with exogenous CCK-8 significantly inhibited morphine dependence, and that activation of central CCK1R by exogenous CCK-8 did attenuate opioid dependence in vivo and in vitro (Wen et al. 2012a, b). Several studies have revealed that the two different CCK receptor subtypes have opposing influences on behavioral actions(Lu et al. 2001; Potter et al. 2012). More evidence is needed to ascertain the role of CCK receptors, especially CCK1R, in the modulation of CCK–opioid system interactions in opioid dependence.

Neuroadaptation in the continual presence of opioids likely involves numerous changes at multiple levels of the signaling pathway. A significant elevation of adenylyl cyclase (AC) activity after drug withdrawal, known as AC supersensitivity or cAMP overshoot, represents an opioid-dependent state in vitro. Moreover, disruption in cell signaling homeostasis leads to the activation of the mitogen-activated protein kinase/extracellular signal-regulated kinase (MAPK/ERK) cascade, as well as certain transcription factors such as cAMP-responsive element binding protein (CREB). Through these transcription factors, transient responses of second messengers become long-lasting changes in gene expression that underlie the neural plasticity and behavioral adaptations seen in opioid dependence.

Herein, we evaluate the effects of CCK1R and CCK2R overexpression on cAMP overshoot in HEK293 cells stably expressing the human µ-opioid receptor (hMOR), with the aim of determining the role of CCK receptor subtypes in opioid dependence. The changes in downstream elements such as ERK and CREB are also evaluated.

## Materials and Methods

### Cell Culture

HEK293 cells (Shanghai Bioleaf Biotec, Shanghai, China) were maintained in high-glucose Dulbecco’s modified Eagle’s medium supplemented with 10% fetal bovine serum (PAA, Pasching, Austria), 100 U/ml penicillin and 100 U/ml streptomycin. All cultures were maintained at 37 °C in a humidified atmosphere of 95% air and 5% CO_2_. For morphine exposure, HEK293 cells was incubated in the serum-free medium with morphine (10 µM), and the medium was replaced every 12 h.

### Stable Cell Transfection and Identification of hMOR

cDNA encoding hMOR (EX-A0868-M02, Genecopoeia, Guangzhou, China) was subcloned into the mammalian expression vector pcDNA™3.1/Hygro(+) (Invitrogen, Carlsbad, California, USA). Transfection of HEK293 cells was performed with Lipofectamine 2000 (Invitrogen) according to the manufacturer’s instructions. Cells transfected with hMOR cDNA were selected with hygromycin B (Roche, Mannheim, Germany) at 100 µg/ml. These HEK293-hMOR cells were cultured in high-glucose DMEM with 10% fetal bovine serum, 100 U/ml penicillin, 100 U/ml streptomycin, and 100 µg/ml hygromycin B in a humidified atmosphere at 5% CO_2_.

[^3^H]-DAMGO binding assays and RT-PCR analysis were adopted to identify the hMOR transfection in HEK293 by compared to non-transfection cell. cDNA was generated using the PrimeScript™ Reverse Transcription System Reagent Kit (Takara Biotechnology, Dalian, China) following the manufacturers’ instructions. In addition [^3^H]-DAMGO binding assays were used to determine the expression level and binding activity of hMOR.

Cells were pelleted and resuspended in ice-cold lysis buffer (50 mM Tris–HCl, 5 mM EDTA, 5 mM EGTA, 1 mM PMSF, pH 7.4). After 30 min, cell suspension was centrifuged at 4 °C and 20,000×*g* for 20 min. The pellets were resuspended in ice-cold 50 mM Tris–HCl buffer (pH 7.4) to pass through the syringe needle and centrifuged again. Membrane preparation was resuspended in TE buffer (5 mM Tris–HCl, 5 mM EDTA, 5 mM EGTA, pH 7.4). The Bradford method was used to determine the protein content. Saturation binding was performed with 0.5–8 nM [^3^H]-DAMGO. Binding was carried out in 5 mM Tris–HCl buffer containing 100 µg membrane preparation with a final volume of 300 µl. Naloxone (10 mM) was used to define specific binding to hMOR. After overnight incubation at 4 °C, bound and free [^3^H]-DAMGO were separated by filtration with grade GF/B filters under reduced pressure, and the filters were washed rapidly three times with 50 mM Tris–HCl buffer (pH 7.4). Radioactivity in the filters was determined by liquid scintillation counting.

### CCK Receptor Overexpression

cDNA encoding CCK1R (EX-A0232-M02, Genecopoeia, Guangzhou, China) or CCK2R (EX-A0966-M02, Genecopoeia) was inserted into a pcDNA™3.1 (Invitrogen) expression vector and transfected into HEK293-hMOR cells using Lipofectamine 2000. CCK receptor expression in HEK293-hMOR cells was measured using real-time PCR. Total RNA was extracted from cell lines using Trizol reagent (Invitrogen). cDNA was generated using the PrimeScript™ Reverse Transcription System Reagent Kit (Takara Biotechnology, Dalian, China) following the manufacturers’ instructions. β-actin was used as the internal control for normalizing total mRNA. All primers (CCK1R: F 5′-GACGCTTCGGTCATTAGA-3′, R 5′-GACGCTTCGGTCATTAGA-3′; CCK2R: F 5′-CCCACTCCCTCCATTGCT-3′, R 5′-CTGCTCCATTCTTATTCCTCTT-3′; β-actin: F 5′-GGGACCTGACTGACTACC TC-3′, R 5′-ACTCGTCATACTCCTGCTTG-3′) were designed in Primer 5.0 and synthesized by Invitrogen (Shanghai, China). The reactions were performed on a 7500 Real-time PCR System (Applied Biosystems, Singapore) for an initial 30 s incubation at 95 °C followed by 40 cycles of 95 °C for 5 s and 60 °C for 35 s. mRNA levels of target genes were calculated using the 2^−ΔΔCt^ method.

### cAMP Accumulation Assays

A LANCE cAMP384 kit (PerkinElmer, MA, USA) was used for cAMP quantification. Cells were seeded onto 24-well plates. The next day, growth medium was replaced with serum-free with 10 µM morphine (Shenyang First Pharmaceutical Factory, China) for 6, 12, 16 or 24 h, then harvested with Versene dissociation solution (Invitrogen) and washed with Hanks’ balanced salt solution (HBSS) 1 × buffer (Invitrogen). The cells were then resuspended at a concentration of 2 × 10^6^ cells/mL in stimulation buffer [HBSS containing 5 mM HEPES (Sigma, St. Louis, MO, USA), 0.1% bovine serum albumin, 0.05 mM IBMX (Sigma)]. Alexa Fluor 647-labeled antibodies were added to the final cell suspension, and then 100 µM naloxone (Sigma) was added to precipitate the cAMP overshoot. After incubation at 37 °C for 15 min, Detection Mix(including in the kit) was added to the mixture. The sample was further incubated for 1 h, and LANCE signal was measured on an Infinite F200 microplate reader (Tecan, Grödig, Austria). A cAMP standard curve was constructed and cellular cAMP level was assayed simultaneously, according to the manufacturer’s instructions. The LANCE signal obtained at 665 nm was used to analyze cAMP levels directly. The signal at 615 nm was used to identify dispensing or quenching problems.

### Measurement of Endogenous CCK

Endogenous CCK content in HEK293-hMOR cells and cell culture supernatant was measured by radioimmunoassay. Sample preparation and CCK assay were performed using a kit (Beijing Sino-uk institute of Biological Technology, China). According to the manufacturer’s instructions, samples were kept at room temperature for 100 min and centrifuged at 4000 r/min for 20 min at 4 °C. 1 mL NaOH (1 mol/L) was added to the supernatant to neutralize the acid. Neutralized samples were centrifuged at 4000 r/min for 10 min and the expression of the and CCK was determined by radioimmunoassay. Results are expressed as pmol/mg protein.

### Western Blotting

The cells cultured as above were used for western blot analysis. After treating with 10 µM morphine for 15 min or 12 h, the cells were lysed in detergent buffer (50 mM Tris–HCl, pH 7.4, 150 mM NaCl, 5 mM EDTA, 10 mM NaF, 10 mM disodium pyrophosphate, 1% Nonidet P-40, 0.5% sodium deoxycholate, 0.1% SDS) in the presence of protease and phosphatase inhibitors. The expression levels of CREB, ERK, p-CREB, p-ERK and were analyzed. Cell debris was centrifuged at 14,000×*g* for 10 min at 4 °C and the supernatant was used for analysis. Proteins were extracted and protein concentration was determined using a BCA protein quantification kit (Solarbio Science & Technology Co., Beijing, China). Total cell lysate was prepared in 1 × SDS buffer and separated by SDS-PAGE. Proteins were then transferred onto a 0.22 µm PVDF membrane (Millipore, CA, USA). The membrane was blocked in 4% non-fat milk, then incubated overnight at 4 °C with antibodies specific for rabbit anti-CREB (1:800; CST, New York, USA), mouse anti-p-CREB (1:500; CST), rabbit anti-ERK (1:800; CST), rabbit anti-p-ERK (1:800; CST) or GAPDH (1:5000; CST). The PVDF membrane was then washed and incubated with IRDye 800CW goat anti-rabbit and goat anti-mouse IgGs (both 1:10,000, Rockland, Florida, USA) for 1 h at 37 °C. The ratios of target protein to internal control were calculated, and the level of target protein was expressed as fold-difference from control. All experiments were performed three times.

### Statistical Analysis

Data were analyzed using SPSS 16.0 and expressed as the mean ± SD of triplicate experiments. Groups were compared by *t* test or one-way ANOVA followed by Tukey’s multiple comparison test, as appropriate. *P* < 0.05 was considered statistically significant.

## Results

### In Vitro Model of Morphine Dependence in HEK293-hMOR Cells

**Fig. 1:**
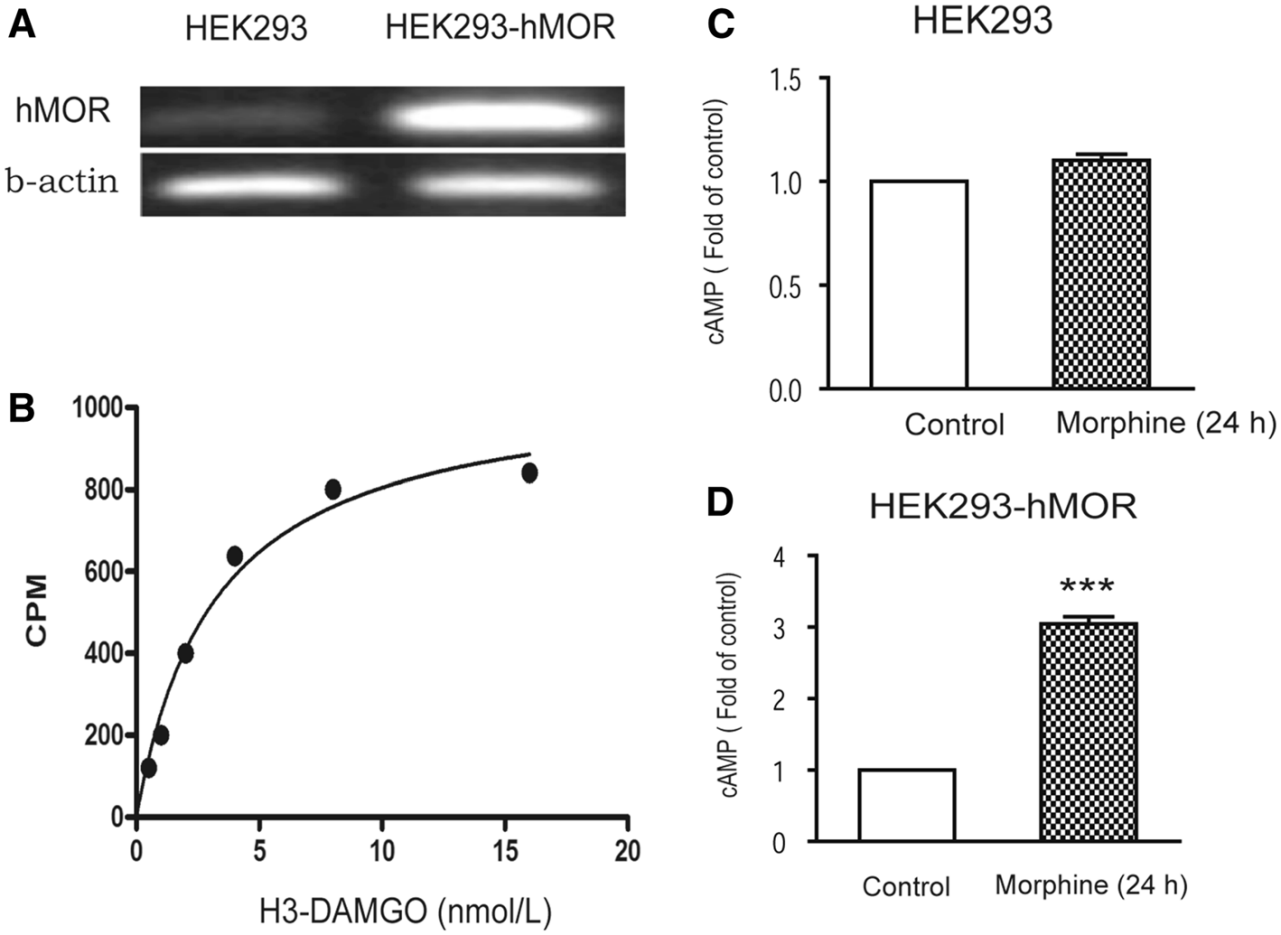
A HEK293 cell line overexpressing human µ-opioid receptors (HEK293-hMOR) was established. [^3^H]-DAMGO binding assays and RT-PCR analysis were adopted to identify the hMOR transfection in HEK293. **a** hMOR expression in transfected cells more than in nontransfected cells by RT-PCR analysis. **b** [^3^H]-DAMGO binding assays showed that in HEK293 hMOR cells, hMOR B_max_ was 1.83 ± 0.13 pmol/mg protein and K_d_ was 0.24 ± 0.02 nM (n = 5). cAMP measurement is to investigate the function and signal transduction pathway of hMOR transfection in HEK293 compared to non-transfection (**c**–**d**). cAMP significantly increased in HEK293-hMOR cells treated by 10 µM morphine for 24 h followed by naloxone (100 µM) precipitation (**d**) but not in HEK293 cells (**c**). Data are presented as the mean ± SEM of three replicates. ***P < 0.001 (vs Control; *t* test)

hMORs were transfected into HEK293 cells, selected using antibiotics, and the transfected and non-transfected cells were cultured for ten passages. The target receptors were stably expressed at high levels (Fig. 1a), with more than 8000-fold greater hMOR expression in transfected cells than in nontransfected cells (*P* < 0.001). Saturation binding assays were performed with [^3^H]-DAMGO to determine the density (B_max_) and affinity (K_d_) of µ-opioid receptors in HEK293-hMOR cells. Untransfected HEK293 cells exhibited no [^3^H]-DAMGO binding (data not shown). In HEK293 hMOR cells, hMOR B_max_ was 1.83 ± 0.13 pmol/mg protein and K_d_ was 0.24 ± 0.02 nM (n = 5; curve fitting by non-linear regression analysis) (Fig. 1b). Exposure to 10 µM morphine for 24 h then treated by 100 µM naloxone for 15 min, the level of cAMP was 3.09 ± 0.289-fold greater than in non-pretreated cells in HEK293 hMOR cells but not non-transfected HEK293 cells (Fig. 1c–d).

### Effect of Chronic Morphine Exposure on the Endogenous CCK System

**Fig. 2:**
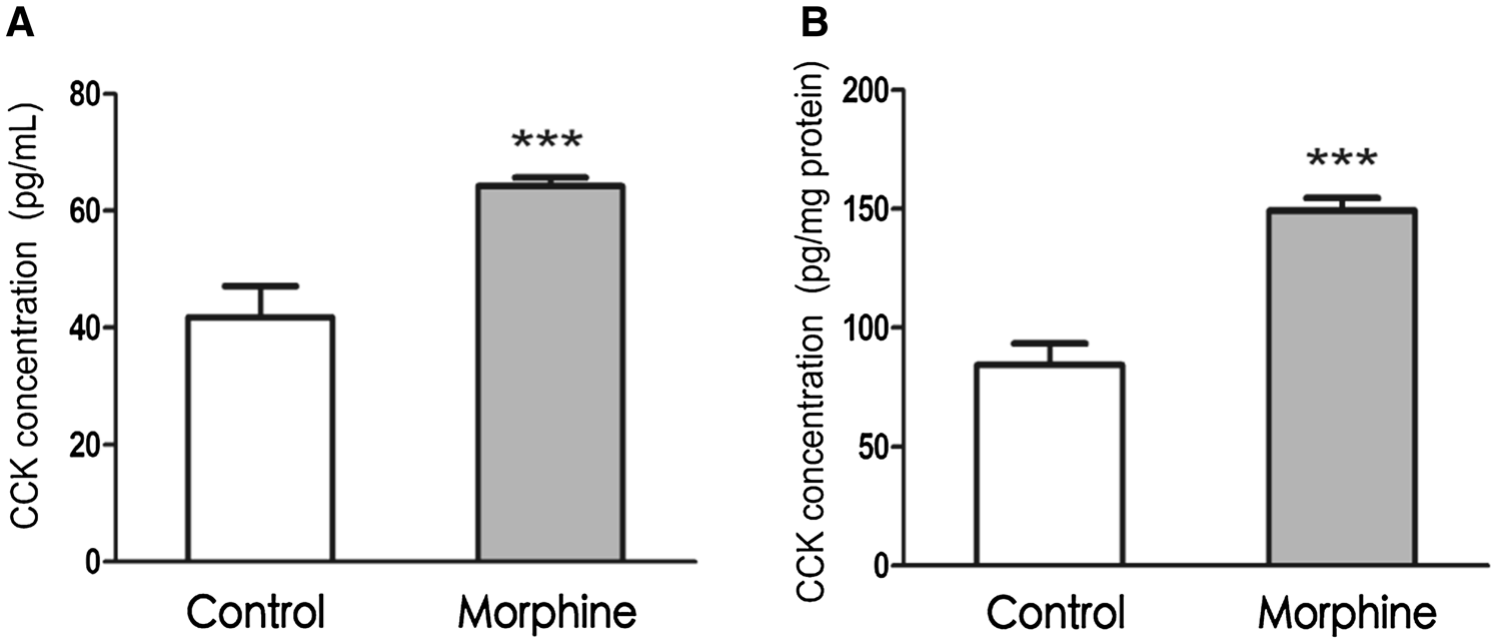
Endogenous CCK content in HEK293-hMOR cells was measured with morphine exposure compared to control group. **a** Endogenous CCK content in cell culture supernatant increased after exposure to 10 µM morphine for 24 h. **b** Endogenous CCK content in HEK293-hMOR cell lysates after exposure to 10 µM morphine for 24 h. Results indicated that not only Endogenous CCK expression but also release were all increased by morphine exposure. Data are presented as the mean ± SEM of three replicates. ***P < 0.001 (vs Control; *t* test)

Endogenous CCK expression in HEK293 cells was confirmed, and the effect of chronic morphine exposure on the accumulation of endogenous CCK in HEK293-Hmor cells was measured. Cell and culture supernatant content of endogenous CCK was upregulated after exposure to 10 Μm morphine for 24 h (Fig. 2a, b).

### Effects of CCK Receptor Overexpression on cAMP System After Morphine Exposure

**Fig. 3:**
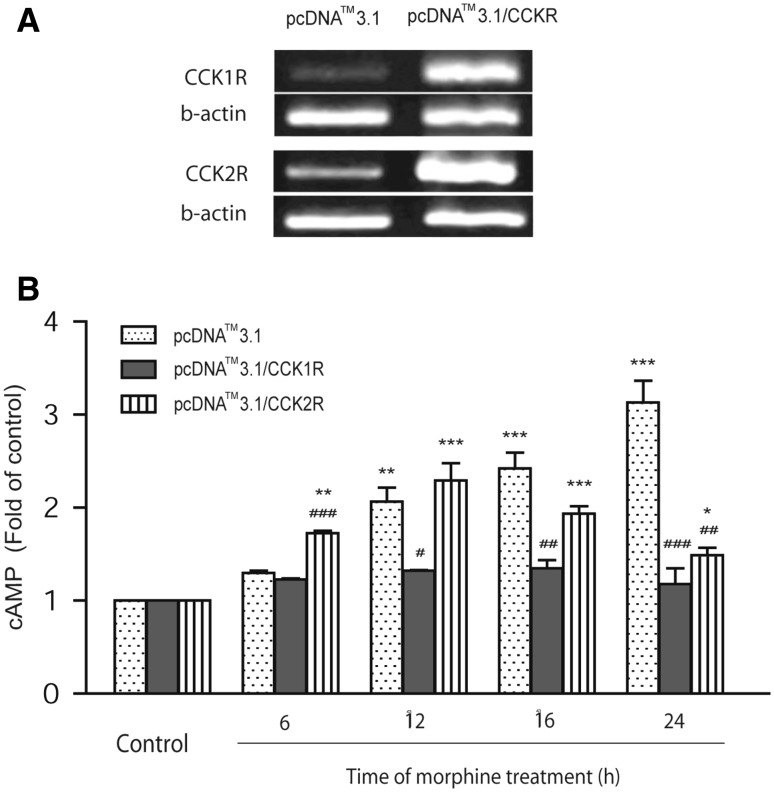
To confirm the CCK receptor overexpression in CCK1R and CCK2R transfected HEK293-hMOR cells, RT-PCR showed CCK1R or CCK2R were increased after transfection (**a**). Then, cAMP assay was adopt to observe effect of CCK1R or CCK2R in morphine induced cAMP overshoot. The concentration of cAMP precipitated by 100 µM naloxone (15 min) in HEK293-hMOR cells at 24 h was approximately threefold greater than in non-treated control cells. The significant increase of cAMP in CCK2R-overexpressing cells was observed after 12 h morphine exposure, but no in CCK1R-overexpressing cells (**b**). Data are presented as mean ± SEM of three replicates. *P < 0.05, **P < 0.01, ***P < 0.001 vs Control; ^#^*P* < 0.05, ^##^*P* < 0.01, ^###^*P* < 0.001 vs HEK293-hMOR (pcDNA^TM^3.1) (one-way ANOVA followed by Tukey’s multiple comparison test)

We explored the effects of CCK receptor overexpression on cAMP changes induced by chronic (6–24 h) exposure to 10 µM morphine. First, CCK1 and CCK2 receptor were transferred to HEK293-hMOR cells (Fig. 3a). In co-transferred HEK293-hMOR cells, naloxone- precipitated cAMP overshoot was observed after 24 h morphine exposure. The concentration of cAMP induced by 100 µM naloxone (15 min) was approximately threefold greater than in non-treated control cells. Furthermore, naloxone-precipitated cAMP overshoot in CCK2R-overexpressing HEK293-hMOR cells was observed after 12 h morphine exposure. However, there was no cAMP overshoot in CCK1R-overexpressing HEK293-hMOR cells (Fig. 3b).

### Effects of CCK Receptor Overexpression on ERK and CREB Phosphorylation After Chronic Morphine Exposure

**Fig. 4:**
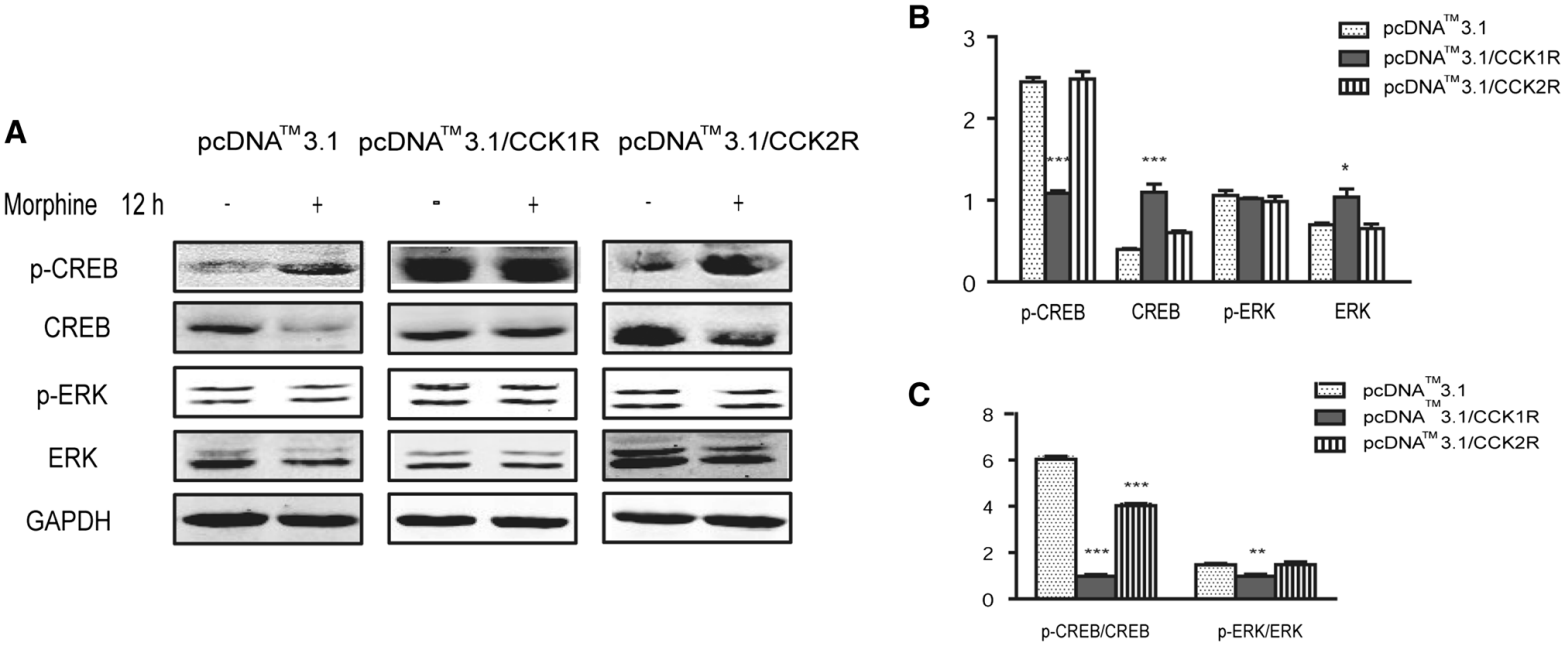
The following experiment design is to confirm CCK1R or CCK2R overexpression on hMOR signal transduction pathway. Here, CCK receptor overexpression on CREB and ERK1/2 activation induced by morphine exposure were observed. The ration of p-CREB/CREB and p-ERK/ERK were significantly increased after 12 h morphine exposure (p-CREB/CREB: 6.20 ± 0.22, p-ERK/ERK: 1.51 ± 0.09 in HEK293-hMOR cells). CCK1R-overexpressing reversed the increased ration of p-CREB/CREB as 1.00 ± 0.13 and p-ERK/ERK as 1.00 ± 0.14 compared to non-transfection cell, but no obvious changes were observed in CCK2R-overexpressing HEK293-hMOR. These data suggest that CCK1R overexpression significantly blocked the effects of chronic morphine exposure on CREB and ERK signaling. Data are presented as the mean ± SD of three replicates. *P < 0.05, **P < 0.01, ***P < 0.001 vs HEK293-hMOR group (pcDNATM3.1) (one-way ANOVA followed by Tukey’s multiple comparison test)

After 12 h morphine exposure, p-CREB/CREB and p-ERK/ERK ratios were significantly higher than in non-exposed control cells (p-CREB/CREB: 6.20 ± 0.22 and p-ERK/ERK: 1.51 ± 0.09 in HEK293-hMOR cells). Results also showed that p-CREB/CREB and p-ERK/ERK ratios were lower in CCK1R-overexpressing HEK293-hMOR than non-transfected HEK293 cells (p-CREB/CREB: 1.00 ± 0.13 and p-ERK/ERK: 1.00 ± 0.14); p-CREB/CREB was also decreased in CCK2R-overexpressing HEK293-hMOR cells (4.14 ± 0.15), but p-ERK/ERK was not obviously changed (1.52 ± 0.18) (Fig. 4). These data suggest that CCK1R overexpression mainly blocked the effects of chronic morphine exposure on CREB and ERK signaling.

## Discussion

Our previous study showed that CCK-8 significantly inhibited cAMP overshoot and measures of naloxone-precipitated withdrawal in vivo and in vitro (Wen et al. 2012b), similar to CCK receptor antagonists. We hypothesized that this phenomenon was related to the different functions of CCK receptor subtypes. Here, we have presented further evidence to demonstrate the different roles of CCK1R and CCK2R in morphine dependence.

HEK293 cells are suitable for expressing hMOR showing ligand-binding selectivity (Arden et al. 1995; Avidor-Reiss et al. 1995; Wu and Wong 2005; Wu et al. 1997). This stable hMOR-transfected cell line provides an excellent model in which to study signal transduction mechanisms and cellular adaptations induced by opioid exposure. In the present study, hMORs were stably expressed at a high level in HEK293 cells, and HEK293-hMOR cells exhibited strong binding affinity for a µ-opioid receptor ligand. Significant changes in cAMP level induced by acute and chronic morphine exposure were elicited in HEK293-hMOR cells. Moreover, the endogenous CCK system in HEK293-hMOR cells was activated by chronic morphine exposure. Thus, this appears to be a useful system in which to study possible interactions between the opioid and CCK systems.

Naloxone-induced withdrawal results in a compensatory increase in AC activity (cAMP overshoot) and is recognized as a sign of opioid dependence(Charles and Hales 2004; Koob and Bloom 1988; Watts 2002). Here, cAMP overshoot in HEK293-hMOR cells was induced by chronic exposure to 10 µM morphine followed by naloxone (100 µM) precipitation, with the peak level elicited after 24 h of morphine exposure, confirming successful establishment of the cellular morphine dependence model. Overexpression of CCK1R significantly inhibited the naloxone-precipitated cAMP overshoot and reduced the compensatory increase cAMP induced by chronic morphine, demonstrating the inhibitory effects of CCK8 on CCK1R in morphine dependence and withdrawal. Compared with HEK293-hMOR cells, cAMP accumulation and cAMP overshoot was elicited earlier in CCK2R-overexpressing HEK293-hMOR cells after chronic (12 h) morphine exposure. Previous studies showed that chronic morphine exposure significantly upregulated the expression of CCK receptors and that CCK2R antagonists block morphine tolerance and morphine-induced conditioned place preference (Lu et al. 2001; Mitchell et al. 2006; Pommier et al. 2002). These data may be explained by endogenous CCK acting on CCK2R, which promotes morphine tolerance and dependence.

Moreover, transcription factors play a crucial role in the development of opioid dependence. CREB is one of the most important factors linking opioid-regulated secondary messenger systems to alterations in gene expression. protein kinase A, CaM kinases or MAPK can phosphorylate CREB at Ser133 to increase its transcriptional activity, and do not respond to the reinforcing properties of morphine in conditioned place preference (Merighi et al. 2013). Recent studies showed that ERK is also involved in morphine dependence (Ren et al. 2013). In cAMP pathway upregulation during chronic exposure to morphine, PKA-dependent ERK phosphorylation may play an important role in MAPK cascade activation. Therefore, ERK, CREB and cAMP second messenger systems transform short-lasting acute opioid signals into long-lasting alterations at the gene transcription level. Our findings demonstrated that chronic morphine exposure significantly activated CREB and ERK signaling in HEK293-hMORcells. In addition, consistent with the results of the cAMP overshoot assay, overexpression of CCK1R blocked the effect of chronic morphine exposure on CREB and ERK signaling, but overexpression of CCK2R did not.
